# Chemical Composition and Antimicrobial Activity of Subcritical CO_2_ Extract of *Lepidium latifolium* L. (Brassicaceae)

**DOI:** 10.1155/2021/4389967

**Published:** 2021-08-04

**Authors:** Balzhan B. Azimkhanova, Gulbaram O. Ustenova, Kamalidin O. Sharipov, Kayrolla D. Rakhimov, Galiya M. Sayakova, Ardak B. Jumagaziyeva, Elena V. Flisyuk, Nadezhda G. Gemejiyeva

**Affiliations:** ^1^Department of Pharmaceutical Technology, Asfendiyarov Kazakh National Medical University, Almaty 050000, Kazakhstan; ^2^Department of Biochemistry, Asfendiyarov Kazakh National Medical University, Almaty 050000, Kazakhstan; ^3^Department of Clinical Pharmacology, Asfendiyarov Kazakh National Medical University, Almaty 050000, Kazakhstan; ^4^Department of Pharmaceutical and Toxicological Chemistry, Pharmacognosy and Botany, Asfendiyarov Kazakh National Medical University, Almaty 050000, Kazakhstan; ^5^Microbiology Laboratory, JSC Scientific Center for Anti-Infectious Drugs, Al-Farabi Ave, 75 A, Almaty 050060, Kazakhstan; ^6^Department Technology of Drugs Forms, Saint-Petersburg State University of Chemical and Pharmaceuticals, St. Petersburg 197376, Russia; ^7^Laboratory of Plant Resources, Institute of Botany and Phyto-Introductions, Almaty 050040, Kazakhstan

## Abstract

The genus *Lepidium* L. from Brassicaceae Burnett. family covers over 150 species with an almost cosmopolitan spread. In Kazakhstan, 21 species are described, of which four species are characterized by medicinal properties (*L. crassifolium* Waldst. et Kit., *L. perfoliatum* L., *L. ruderale* L., and *L. latifolium* L.), used in folk medicine as means of antibacterial, irritant, laxative, antitumor, analgesic, and anthelmintic action. *Methods*. Raw materials were collected from Almaty region (Republic of Kazakhstan). *Lepidium latifolium L.* herb's carbon dioxide extract (CO_2_ extract) was obtained by subcritical carbon dioxide extraction. A gas chromatograph with a mass spectrometric detector was used to determine the component composition of the extract. Antimicrobial activity was determined by two methods: the micromethod of serial dilution and the disc-diffusion method. Four microbial test strains were used: *Staphylococcus aureus* ATCC 6538-P, *Escherichia coli* ATCC 8739, *Klebsiella pneumonia* ATCC 10031, and *Candida albicans* ATCC 10231. *Results*. The technology of carbon dioxide extraction has undoubted advantages over traditional methods of extraction: it has a controlled selectivity in relation to groups of biologically active substances, allows deep extraction, and maximizes the release of rich complexes of compounds contained in plants. In this study, firstly, the CO_2_ extract was obtained under subcritical conditions from the aerial part of *L. latifolium* L., and the composition was determined. Hexane was the best solvent for CO_2_ extract, and 40 components were identified. Screening of antimicrobial activity of the *L. latifolium*'s CO_2_ extract showed the essential activity of all clinically significant strains tested: *Staphylococcus aureus*, *Escherichia coli*, *Klebsiella pneumonia*, and *Candida albicans*. *Conclusions*. This research showed that the CO_2_ extract of the raw material of *Lepidium latifolium* L. contains biologically active compounds exhibiting an essential antimicrobial effect, and therefore it is possible to recommend for the development of various drugs for use in medical practice.

## 1. Introduction

Medicinal plants have been used since ancient times for their medicinal value. They are a valuable source of biologically active substances with very different biological and pharmacological activities [1]. Herbal remedies are characterized by minimal price in comparison with synthetic drugs, easy availability, relative safety, and low toxicity, and they also act in a complex way, which allows them to be used for prevention and long-term treatment of different diseases [2].

*Lepidium* L. gen. encompasses over 150 species with an almost cosmopolitan spread. In Kazakhstan, there are 21 species with 3 endemics: *L. karataviense* Regel et Schmalh., *L. rubzovii* Vassil., and *L. alberti* Pavl. [3].

There are four species characterized by medicinal properties (*L. crassifolium* Waldst. et Kit., *L. perfoliatum* L., *L. ruderale* L., and *L. latifolium* L.) and used in folk medicine as antibacterial, irritant, laxative, antitumor, analgesic, and anthelmintic. Containing saponins, flavonoids, alkaloids, glycosides, and tannins [4], the leaves have the following steroids: cholesterol, stigmasterol, and beta-sitosterol [5].

*Lepidium latifolium* L. is a promising medicinal plant, a perennial herb 30–100 (150) cm high, slightly pubescent or bare. The root is up to 3–12 mm thick. The stem is straight and branched in the upper part. The basal leaves are rigid, elliptical or ovate-lanceolate, and acute; the upper leaves are lanceolate, small, and almost sessile. The flowers are white and small and collected in a pyramidal paniculate inflorescence. The pods are ovoid, without a notch at the apex. The seeds are broadly elliptical, almost smooth. Blossoms and bears fruit in May–August. In Kazakhstan, *Lepidium latifolium* L. can be found everywhere—from the plains to the mid-mountain zone—in meadows, salt marshes, saline places in the steppe, humid places, along river valleys, as a weed in crops, and near dwellings [6].

General spread occurs in Europe, Mediterranean countries, Central and Western Asia, Pakistan, Russia, Tibet, Himalayas, Western and Eastern China, Mongolia, North and Central America, and Australia [7].

*L. latifolium* has been reported as an invasive species in Montana (USA) [8].

According to the literature, *L. latifolium* is used as a herbal product, side dish, drink, and also a herbaceous medicinal plant with anti-inflammatory, antibacterial, diuretic, and tonic action [9]. The Western Himalayan ecotype of this plant is used as a herbal product for the treatment of the gastrointestinal tract. In folk medicine, decoction and infusion of herbs and roots are used for skin diseases, wounds, pain in joints, scurvy, toothache, ascites, and disorders of the nervous and digestive systems [10].

The oxidative stress mediated by free radicals is one of the main causes of many chronic diseases and diabetes. The ethyl acetate-soluble *L. latifolium* fraction is a rich source of antioxidants for the treatment of a number of diseases associated with oxidative byproducts of human metabolism due to its high content of phenolic compounds having powerful ability to clear free radicals. It is also a potential natural source of antioxidants for the treatment of some diseases associated with oxidative products of human metabolism [11].

Analysis of the fatty acids composition demonstrated that the leaves of the species examined are rich in polyunsaturated fatty acids, in particular, linoleic and linolenoic acids, with their content reaching 50%. The high glucose and protein content, along with the high nitrogen to sulphur ratio, also add to the nutritional value of this plant [12].

The leaves and flowers of *L. latifolium* contain kaempferol-3-d-glucofuranosyl-6-L-rhamnopyranoside and quercetin-3-d-glucopyranoside flavonoids. They are consumed daily in powder form with water to treat joint pain in rheumatism [13]. Sulphur essences have been identified in dry leaves of *L. latifolium*, which are powerful means for the destruction of kidney stones and other obstructions of the urinary tract underlining their effective diuretic action [14].

*L. latifolium* contains flavonoids with antioxidant, anti-inflammatory, and steroidal activity and has an inhibitory effect of aromatase [15].

*L. latifolium* extract exhibits cytotoxic activity *in vitro*, inducing caspase-dependent apoptosis in various human tumor cells. Epithionitrile-1-cyano-2,3-epithiopropane (CETP) was identified as the main factor of *L. latifolium*, which actively kills cancer cells [16].

The cultivated perennial plant of *L. latifolium* contains thioglycosides, of which sinigrin and glucotropaeolin are the most common. The anticarcinogenic and antimicrobial activities of the obtained extract against pathogenic bacteria and fungi were also evaluated [17]. Therefore, *L. latifolium* is very promising for further phytochemical study of its composition and therapeutic action.

The technology of carbon dioxide extraction has undoubted advantages over traditional methods of extraction: it has a controlled selectivity in relation to groups of biologically active substances, allows deep extraction, and maximizes the release of rich complexes of compounds contained in plants. Advantages of subcritical carbon dioxide extraction compared to supercritical extraction are as follows: the process is more cost-effective and more technologically advanced and allows for a clean final product [18].

In this study, we obtained the thick CO_2_ extract in the subcritical conditions from the aboveground part of *Lepidium latifolium* L*.,* explored component composition depending on the solvent, and established its antimicrobial activity against pathogenic microorganisms for the first time. The results of this study make it possible to use the CO_2_ extract of *Lepidium latifolium* L. to develop antibacterial drugs and cosmetic products for medical practice.

## 2. Materials and Methods

### 2.1. Plant Material

The material for the research was the aerial part of the flowering *Lepidium latifolium* L., collected in June–July 2018 in the Sogety mountains (north-eastern spur of the Zailiyskiy Alatau ridge) on the territory of the Enbekshikazakhsky district of Almaty region (Republic of Kazakhstan) (43°33.304′ N, 078°25.748′ E). The plant material was collected at 10 : 00 am on a bright sunny day, and grass was dried at a temperature of 25°C.

The drying of raw materials was carried out in a well-ventilated room at a temperature of +25 ± 5°C. The moisture content of the raw material was ±6.35%.

The plant was identified by the Institute of Botany and Phytointroduction of the Forestry and Wildlife Committee of the Ministry of Ecology, Geology and Natural Resources of the Republic of Kazakhstan.

### 2.2. Preparation of the Extract

*Lepidium latifolium* L. herb's carbon dioxide extract was obtained from the aboveground part of the raw material, and the extraction was carried out under subcritical conditions on a laboratory extraction unit (installation of carbon dioxide flow-through extraction-5L) using liquid CO_2_ as an extractant. The optimal conditions for obtaining subcritical CO_2_ extract were as follows: pressure 45–51 atm, temperature 18–21°C, and extraction time 11 hours. Raw materials were crushed on a KDU-2 crusher to the size of 1–3 mm. 2.6 kg of the raw material was used, while the extract yield was 35 g (1.35%). In appearance, the thick subcritical CO_2_ extract from the *Lepidium latifolium* L. is brown colored and possesses a specific smell.

The use of subcritical extraction increases the biological value and safety of the extracted substances, while supercritical carbon dioxide extraction negatively affects the safety of the extracted substances–some of the thermolabile compounds disintegrate, thereby violating the integrity of the extraction and at the same time polluting the final product [19].

To determine the component composition, the thick carbon dioxide extract was dissolved in the following solvents: hexane (99.6%, Sigma-Aldrich), methanol (99.9%, Sigma-Aldrich), ethyl acetate (99.8%, AppliChem GmbH), and dichloromethane (99.9%, Sigma-Aldrich). 0.1 grams of the test extract was dissolved in 10 ml of these solutions (0.01 g/ml).

### 2.3. Component Composition Determination and Component Identification

The analysis was carried out by gas chromatograph with an Agilent 7890B/5977A mass spectrometric detector (GC-MS). The chromatographic analysis conditions were as follows: sample volume: 0.2 *μ*l and sample injection temperature: 240°С, without dividing the flow. The separation was carried out using a WAXetr chromatographic capillary column of a 30 m length, 0.25 mm inner diameter, and 0.25 *μ*m film thickness at a constant carrier gas (helium) velocity of 1 ml/min. The chromatographic temperature was programmed from 40°C (0 min exposure) to 260°C with a heating rate of 10°C/min (20 min exposure). The detection was carried out in the SCAN m/z 34–850 mode. Agilent MSD Chem Station software (1701EA version) was used for controlling the gas chromatography system, registering and processing the obtained results and data. Data processing included determination of determent time intervals and peak areas, as well as processing of spectral information obtained using the mass spectrometric detector. The Wiley 7th edition and NIST'02 libraries were used to identify the mass spectra obtained.

### 2.4. Investigation of Antimicrobial Activity

Four microorganisms strains were tested: Gram-positive bacteria (*Staphylococcus aureus* ATCC 6538-P), Gram-negative bacteria (*Klebsiella pneumonia* ATCC 10031, *Escherichia coli* ATCC 8739), and *Candida albicans* ATCC 10231.

Sensitivity studies of microorganisms were performed on standard nutrient media:

  Mueller Hinton Agar (М173), HiMedia, India
  Mueller Hinton Broth (М391), HiMedia, India
  Fluid Sabouraud medium (M013), HiMedia, India

To determine the antimicrobial activity, thick CO_2_ extract was dissolved in hexane.

To prepare suspensions of microorganisms of the desired concentration, a DEN-1 densitometer was used to measure the optical density (turbidity). Suspensions of microorganisms were prepared on a saline solution of sodium chloride (0.9% NaCl). 5 ml of saline solution was added to the test tube, which was placed in a densitometer, and the optical density was measured. First, a suspension of microorganisms with a concentration of 1.5 × 10^8^ CFU/ml for bacteria was prepared, which corresponds to turbidity of 0.5 units according to McFarland; tenfold dilutions were made from these suspensions, transferring 1.0 ml of the suspension in 9.0 ml of sterile saline solution. Thus, a dilution of 1.5 × 10^6^ CFU/ml for bacteria was obtained. For mushrooms, the suspension was prepared in the same way. The density of the fungi suspension corresponded to 2.0 × 10^6^ CFU/ml.

#### 2.4.1. Serial Dilution Method

A 96-well plate was used to determine the antimicrobial activity [20, 21]. In all wells, except for the first ones, the Mueller-Hinton nutrient broth (for testing bacteria) and Sabouraud-dextrose broth (for testing fungi) were added in the amount of 100 *μ*l. The extract was introduced in the volume of 100 *μ*l into the 1st well, making serial dilutions by taking the mixture (Muller-Hinton broth/Sabouraud-dextrose broth (100 *μ*l) + test drug (100 *μ*l)) from the 1st well in the amount of 100 *μ*l into the 2nd well, already containing 100 *μ*l of the broth. The test sample was thoroughly mixed and then transferred in the amount of 100 *μ*l into the broth from the 2nd well to the 3rd well, which also initially contained 100 *μ*l of the broth. This procedure was repeated until the required number of dilutions was reached.

After a series of dilutions, 20 *μ*l of test strains of microorganisms was added to all wells. The seeded plates were incubated for 18–24 hours at a temperature of 37 ± 1°C. At the end of the incubation time, 0.01 ml per Petri dish with agarized medium was seeded from each well using a sterile loop. After seeding, the cups were placed in a thermostat for 18–24 hours at 37 ± 1°C.

The results were considered by the presence of visible growth of microorganisms on the surface of the dense nutrient medium. The minimum bactericidal/fungicidal concentration (MBC/MFC) was considered the lowest concentration in the test well that suppressed the growth of microorganisms.

#### 2.4.2. Disc-Diffusion Method

Petri dishes on the entire surface of Muller-Hinton agar were preseeded with a suspension of microorganisms with a density of 1.5 × 10^8^ CFU/ml, and a suspension of *Candida albicans* at a concentration of 7.5 × 10^8^ CFU/ml was seeded with cups on the entire surface of Saburo agar [22, 23]. Next, the discs, preimpregnated with the studied concentration of hexane solution of carbon dioxide extract (1000 *μ*g/ml), were applied to the surface of the inoculated culture. Hexane was tested as a control sample. Discs of ampicillin (10 *μ*g/disc) were used as standards for all bacteria. Fluconazole (25 *μ*g/disc) discs were used for *C. albicans*. The samples were placed in a thermostat for incubation for 18–24 hours at 37°C for bacteria, and *Candida albicans* was incubated at 22°C for 48 hours. The results were taken by calculating the diameter of the growth delay/suppression zones with an accuracy of 1 mm.

## 3. Results and Discussion

### 3.1. Compounds Identified in the CO_2_ Extract of *Lepidium latifolium* L

**Figure 1:**
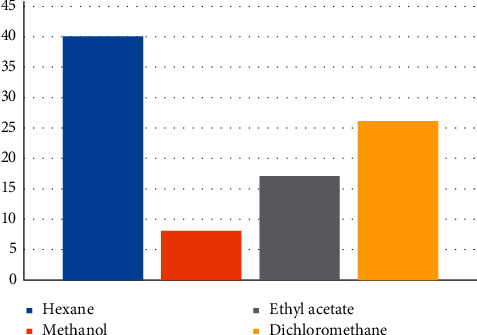
The quantitative content of the biologically active substances of subcritical СО_2_ extract of *Lepidium latifolium* L. depending on the solvent (the OY axis is the amount of biologically active compounds, and the OX axis is the solvent of the СО_2_ extract).

In this work, the phytochemical composition of the subcritical CO_2_ extract of *Lepidium latifolium* L. was determined using organic solvents: hexane, methanol, ethyl acetate, and dichloromethane. The GC/MS screening has revealed that hexane is the optimal solvent for the carbon dioxide extract. As shown in Figure 1, the highest yield of biologically active substances was observed in hexane solution (40 compounds), while 8 compounds were identified in methanol solution, and 17 and 26 in ethyl acetate and dichloromethane solutions, respectively.

**Figure 2:**
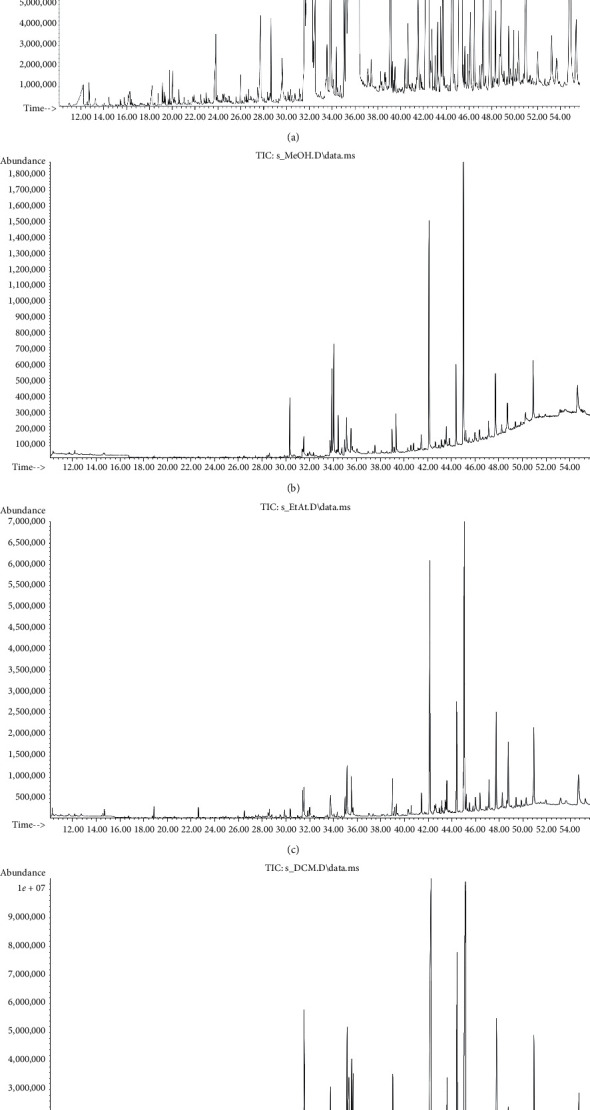
Typical GC-MS chromatograms of hexane (a), methanol (b), ethyl acetate (c), and dichloromethane (d) solutions of CO_2_ extract of *Lepidium latifolium* L.

The main ingredients which were found in the subcritical СО_2_ extract were phytosterols such as *β*-sitosterol, campesterol, and stigmasterol and diterpenes such as phytol, vitamin E, and fatty acids (Table 1, Figure 2). They indicate significant specific pharmacological activity, in particular antibacterial, anti-inflammatory, antitumor, and antioxidant [24–27]. In quantitative terms, the content of these substances dominated in the hexane solution: *β*-sitosterol (12.71%), campesterol (2.8%), stigmasterol (1.76%), phytol (7.3%), and vitamin E (10.84%). All these compounds, except stigmasterol, were also observed in other tested solutions; for example, the content of *β*-sitosterol in methanol solution was 11.8% and in ethyl acetate and dichloromethane solutions was 9.6% and 6.12%, and the content of campesterol in the ethyl acetate and dichloromethane solutions was 1.9% and 1.21%, while it was not identified in the methanol solution. The phytol content in the methanol solution was 7.3%, and in the ethyl acetate and dichloromethane solutions its content was 3.6% and 3.9%, respectively.

It is worth noting that *β*-sitosterol has antibacterial potential. When studying against *Salmonella typhi* and *Escherichia coli* strains, the values of growth inhibition zones were 20 and 35 mm, respectively [28]. The activity of a *β*-sitosterol chloroform extract of *M. parviflora* roots against *S. aureus* and *E. coli* was studied [29]. It has been confirmed that *β*-sitosterol has a significant anti-inflammatory effect and, as one of the most important herbal components, has the potential of an anti-inflammatory drug due to its wide source and nontoxic natural properties [30]. Stigmasterol exhibits antibacterial activity against methicillin-resistant *Staphylococcus aureus* [31], and it has been shown to significantly inhibit tumor progression in two-stage carcinogenesis [32]. The extract of phytosterols (*β*-sitosterol, campesterol, stigmasterol, and lanosterol) of *Datura stramonium* showed a strong antimicrobial activity against *P. aeruginosa* (22.2 ± 0.59 mm) and *A. niger* (14.5 ± 0.25 mm) [33].

Recent studies with phytol demonstrated anxiolytic, metabolism modulating, cytotoxic, antioxidant, inducing autophagy and apoptosis, anti-inflammatory, immunomodulatory, and antimicrobial effects [34].

### 3.2. Results of Antimicrobial Activity

When determining the antimicrobial activity both by the method of serial dilutions and by the disc-diffusion method, the antibacterial and antifungicidal activities of the hexane solution of the carbon dioxide extract of *Lepidium latifolium* were established in relation to the analyzed strains of microorganisms—*S. aureus, E. coli, Kl. pneumonia*, and *C. albicans* (Tables 2 and 3). Previous studies confirmed the antimicrobial activity of *Lepidium latifolium.* The dichloromethane extract and allyl isothiocyanate (degradation product of glucosinolate) of *L. latifolium* have significant inhibitory activity against bacteria, such as *Staphylococcus aureus*, *Escherichia coli*, *Pseudomonas aeruginosa*, and *C. albicans* [35]. Compounds with phenolic hydroxyl groups isolated from an ethanol extract of *L. latifolium* exhibit significant antibacterial activity against four strains: *Bacillus subtilis, Staphylococcus aureus, Escherichia coli*, and *Pseudomonas aeruginosa* [36].

**Figure 3:**
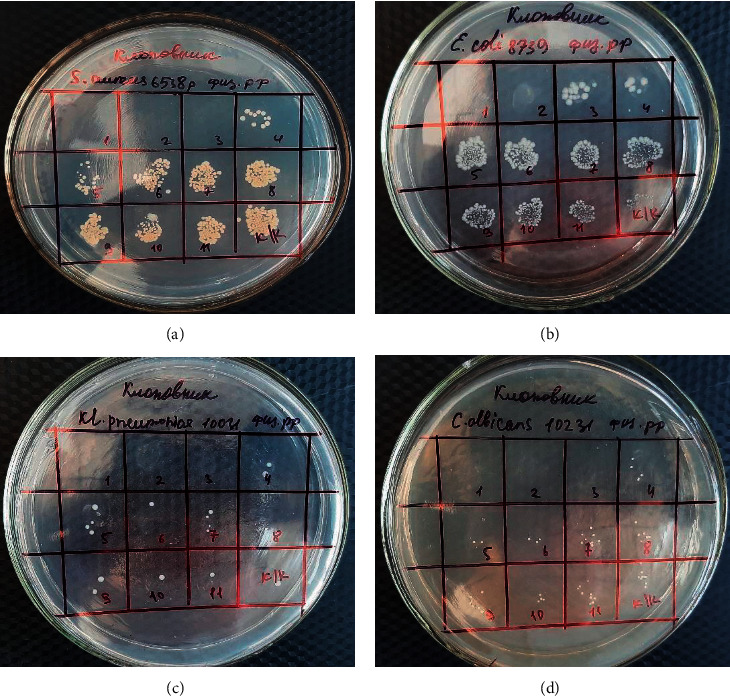
Results of the antimicrobial activity of *Lepidium latifolium*'*s* CO_2_ extract obtained by the serial dilution method: (a) *S*. *aureus*; (b) *E*. *coli*; (c) *Kl. pneumonia*; (d) *C*. *albicans*.

The results of the research of antimicrobial activity by serial dilution showed that the hexane solution of subcritical CO_2_ extract of *Lepidium latifolium* L. had the highest antimicrobial activity against *S. aureus*, *Kl. pneumonia*, and *C. albicans* at a concentration of 32 *μ*g/ml; against *E. coli*, it had an established bactericidal activity at a concentration of 125 *μ*g/ml (Figure 3).

**Figure 4:**
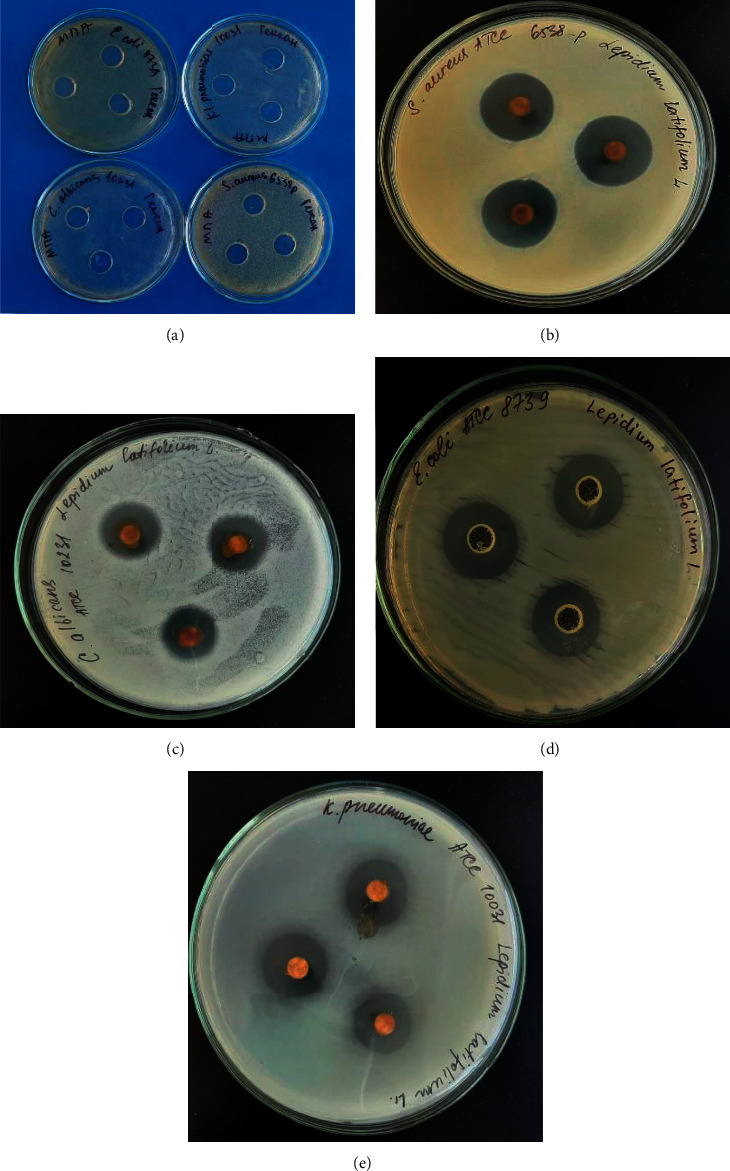
Results of the antimicrobial activity of *Lepidium latifolium*'*s* CO_2_ extract obtained by the disc-diffusion method: (a) Control sample (hexane); (b) *S*. *aureus*; (c) *C*. *albicans*; (d) *E*. *coli*; (e) *Kl. pneumonia.*

When studying the effectiveness of a hexane solution of subcritical CO_2_ extract of *Lepidium latifolium* L*.,* the disc-diffusion method was used to obtain data with high values of the growth suppression zone in comparison with the used standards of antibiotics. The growth delay zones of the test strains were 18.33 ± 0.57 against *Kl. pneumonia*, 17.1 ± 1.0 against *E. coli*, and 20.2 ± 1.0 against *S. aureus*. Antifungicidal activity was also established against *C. albicans* with a growth retardation zone reaching 19.33 ± 0.57 mm. Growth retardation zone against *S. aureus*, *E. coli*, *Kl. pneumonia*, and *C. albicans* strains, with the action of antibiotics, was 15.6 ± 0.57 mm, 12.3 ± 0.57 mm, 12.3 ± 0.57 mm, and 14.0 ± 0.0 mm, respectively. It was also found hexane as a control does not show antimicrobial activity against cultures of microorganisms (Figure 4). It was conditionally accepted that the diameter of the growth zone delay was over 15 mm for high activity, 10–15 mm for medium activity, and less than 10 mm for low activity [37].

In recent years, human pathogens have acquired resistance to the synthetic antibiotics used, which leads to an increase in the severity of infectious diseases. The unwanted side effects of some antibiotics and the appearance of rare infections have prompted scientists to look for new antimicrobial agents. Screening of plant extracts makes them a potential source of antimicrobial agents [38].

## 4. Conclusion

А CO_2_ extract from the aerial part of the *Lepidium latifolium* L. crude drug was obtained, and its chemical composition was determined. Of all the solvents tested, hexane is optimal for the dissolution of the extract, because the hexane solution was distinguished by a richer set of biologically active substances. The main components are phytosterols, diterpenes, vitamin E, and fatty acids.

In all solutions of the carbon dioxide extract, the predominant component was *β*-sitosterol with its content in the hexane solution reaching 12.71% and in methanol, ethyl acetate, and dichloromethane solutions being 11.8%, 9.6%, and 6.12%, respectively. Campesterol was present in all solutions except that of methanol, while stigmasterol was identified only in hexane solution at 1.76%. Vitamin E was present in all solutions, except that of ethyl acetate; its maximum yield was recorded in methanol solution. According to the scientific literature, the presented biologically active substances have very valuable pharmacological actions, such as antimicrobial, anti-inflammatory, antitumor, etc.

The results of the antimicrobial activity screening showed that the hexane solution of CO_2_ extract of *Lepidium latifolium* L. exhibits antimicrobial activity against all the test strains (*S. aureus*, *E. coli*, *Kl. pneumonia*, and *C. albicans*) by the method of serial dilutions and by diffusion in agar.

Thus, for the first time, the carbon dioxide extract was obtained under subcritical conditions from the aerial part of the *Lepidium latifolium* L. crude drug, and its phytochemical composition was determined using various solvents. The results obtained indicate that hexane is the optimal solvent for the extract. A large number of *β*-sitosterol, campesterol, stigmasterol, phytol, and vitamin E were identified. It was found that the hexane solution of CO_2_ extract had an antimicrobial effect, which was confirmed *in vitro*.

The results of this study make it possible to use the CO_2_ extract of *Lepidium latifolium* L. to develop antibacterial drugs and cosmetic products for medical practice. Research in this direction is being continued.

## Figures and Tables

**Table 1:** The results of compound components of the hexane, methanol, ethyl acetate, and dichloromethane solutions of subcritical CO_2_ extract of *Lepidium latifolium* L.

No.	Retention time, min	Compound name	Content (%)			
			Hexane	Methanol	Ethyl acetate	Dichloromethane
1	10.3	Cyclohexanone	—	—	1.2	—
2	12.7	2,4-Heptadienal	—	—	—	0.21
3	14.7	1-Dodecene	—	—	0.7	—
4	16.3	Octanoic acid	0.56	—	—	—
5	18.2	Nonanoic acid	0.89	—	—	0.36
6	18.9	1-Tetradecene	—	—	1.0	—
7	19.1	2,4-Decadienal	0.41	—	—	—
8	20.0	Decanoic acid	0.88	—	—	—
9	20.6	Caryophyllene	0.27	—	—	—
10	21.0	Undecanoic acid	0.24	—	—	—
11	21.9	5,9-Undecadien-2-one, 6,10-dimethyl-	0.23	—	—	—
12	22.5	Hexadecane	0.20	—	1.1	—
13	23.8	Dodecanoic acid	2.49	—	—	—
14	24.0	Nonanoic acid, 9-oxo-, ethyl ester	0.19	—	—	—
15	24.4	Heptadecane	0.22	—	—	—
16	26.0	2(4H)-Benzofuranone, 5,6,7,7a-tetrahydro-4,4,7a-trimethyl-	0.50	—	—	0.27
17	27.4	3,7,11,15-Tetramethyl-2-hexadecen-1-ol	0.27	—	—	—
18	27.7	Tetradecanoic acid	3.33	—	—	0.44
19	28.3	Nonadecane	0.21	—	—	—
20	28.5	3-Buten-2-one, 4-(4-hydroxy-2,2,6-trimethyl-7-oxabicyclo[4.1.0]hept-1-yl)-	—	—	—	0.21
21	28.6	2-Pentadecanone, 6,10,14-trimethyl-	1.77	—	1.0	0.63
22	29.6	Pentadecanoic acid	2.32	—	—	0.20
23	29.9	Benzoic acid, hept-2-yl ester	—	—	0.7	—
24	30.3	Hexadecanoic acid, methyl ester	0.24	—	3.4	—
25	30.4	Benzoic acid, pentadecyl ester	—	—	1.5	—
26	30.7	1,4-Naphthalenedione, 2,3,6-trimethyl-	0.31	—	—	—
27	31.1	5,9,13-Pentadecatrien-2-one, 6,10,14-trimethyl-	0.33	—	—	—
28	31.2	Benzoic acid, 4-hydroxy-3,5-dimethoxy-, hydrazide	0.22	—	—	—
29	31.5	Hexadecanoic acid, ethyl ester	—	—	—	12.07
30	31.9	Methyl 8,11,14-heptadecatrienoate	—	—	0.8	—
31	33.6	Phthalic acid, butyl 8-methylnonyl ester	3.11	—	—	—
32	33.9	Phytol	7.30	3.6	3.9	4.12
33	34.4	p-Octylacetophenone	1.21	—	—	0.68
34	35.1	Ethyl oleate	3.53	2.7	2.6	1.93
35	38.1	Hexadecanoic acid, 1-(hydroxymethyl)-1,2-ethanediyl ester	—	—	—	0.17
36	38.6	Methyl 19-methyl-eicosanoate	1.66	—	—	0.37
37	39.0	Hexacosane	—	—	—	3.49
38	39.3	4,8,12,16-Tetramethylheptadecan-4-olide	0.94	—	—	0.53
39	41.4	Octadecane, 3-ethyl-5-(2-ethylbutyl)-	—	3.3	—	—
40	41.5	Octacosane	4.47	53.4	37.8	3.49
41	41.8	Docosanoic acid, ethyl ester	0.60	—	—	—
42	42.6	13-Methylheptacosane	0.98	—	—	—
43	44.4	Nonacosane	—	—	13.8	23.71
44	44.6	Hexacosane, 9-octyl-	9.95	4.1	4.3	1.12
45	45.2	Squalene	—	—	—	1.54
46	46.5	Triacontane	2.39	—	—	22.33
47	47.9	Tetratriacontane	8.24	—	—	6.65
48	48.3	Octadecanal	2.15	—	—	—
49	48.7	Hentriacontane	—	7.3	—	—
50	49.9	*γ*-Tocopherol	1.58	—	10.7	0.88
51	51.0	Vitamin E	10.84	13.8	—	5.71
52	52.0	Phytol acetate	1.74	—	—	—
53	53.2	Campesterol	2.80	—	1.9	1.21
54	53.7	Stigmasterol	1.76	—	—	—
55	54.9	*β*-Sitosterol	12.71	11.8	9.6	6.12
56	55.4	Phytol, acetate	2.85	—	—	1.04

**Table 2:** The results of the antimicrobial activity of *Lepidium latifolium*'*s* hexane solution of subcritical CO_2_ extract obtained by the method of serial dilutions.

Test sample	MBC/MFC (*μ*g/ml)			
	*S. aureus* ATCC 6538-Р	*E. coli* ATCC 8739	*Kl. pneumonia* ATCC 10031	*C. albicans* ATCC 10231
*Lepidium latifolium*'*s* hexane solution of subcritical CO_2_ extract	32	125	32	32

**Table 3:** The results of the antimicrobial activity of *Lepidium latifolium*'*s* hexane solution of subcritical CO_2_ extract obtained by the disc-diffusion method.

Test sample	Growth suppression zone (mm)			
	*S. aureus* ATCC 6538-Р	*E. coli* ATCC 8739	*Kl. pneumonia* ATCC 10031	*C. albicans* ATCC 10231
Control sample (hexane)	0	0	0	0
*Lepidium latifolium's* hexane solution of subcritical CO_2_ extract, 1000 *μ*g/ml	20.2 ± 1.0	17.1 ± 1.0	18.33 ± 0.57	19.33 ± 0.57
^*∗*^Antibiotic	15.6 ± 0.57	12.3 ± 0.57	12.3 ± 0.57	14.0 ± 0.0

^*∗*^Discs of ampicillin (10 *μ*g/disc) were used as standards for all bacteria. Fluconazole (25 *μ*g/disc) discs were used for *C. albicans.*

## Data Availability

The data used to support the findings of this study are available from the corresponding author upon request.
